# Bergaptol Ameliorates Insulin Sensitivity in Gestational Diabetes Mellitus by Inhibiting the Inflammatory Pathway in Streptozotocin-Induced Diabetic Rats

**DOI:** 10.33549/physiolres.935474

**Published:** 2025-02-01

**Authors:** Qianrong LI, Jin CHEN, Xiaoyin WANG, Lin ZHUANG, Zhi YU, Dan YANG

**Affiliations:** 1 Department of Obstetrics, Hospital of Chengdu University of Traditional Chinese Medicine, Chengdu, China

**Keywords:** Gestational diabetes, Bergaptol, Insulin resistance, Inflammation, Oxidative stress

## Abstract

Investigation determines the beneficial effect of bergaptol against gestational diabetes (GD). Gestational diabetes was induced in female rats and treated them with bergaptol 20 and 40 mg/kg for eighteen days. Effect of bergaptol was assessed on blood glucose and insulin level in GD rat. Inflammatory mediators and oxidative stress parameters were also assessed in GD rats. Moreover, mRNA expression of INSR, NF-κB, Akt and GSK-3β were assessed in the GD rats by qRT-PCR method. *In silico* network pharmacology study was performed, along with gene ontology and egg pathway to assessed the targets of bergaptol, molecular docking study was also performed for the confirmation of possible pathway involved in the management of GD. Blood glucose and insulin level was significantly reduces in the blood bergaptol treated group than GD group of rats. Treatment with bergaptol ameliorates the altered level of mediators of inflammation and oxidative stress parameters in GD rats. There was significant reduction in the mRNA expression of NF-κB and GSK-3β and increase in expression of INSR and Akt in the tissue homogenate of bergaptol treated GD rats. Docking study shows effective binding strength of bergaptol individually with INSR, NF-κB, Akt and GSK-3β-protein targets. In conclusion, data of investigation suggest that bergaptol improves the sensitivity of insulin receptor in GD, as it reduces parameters of oxidative stress and inflammatory mediators by regulating INSR/NF-κB/Akt/GSK-3β pathway.

## Introduction

Gestational diabetes (GD) is a type of diabetes, insulin resistance occur at the time of pregnancy, higher prevalence rate with mortality [1]. However pathogenesis of GD is yet to be fully understood. Insulin resistance develop due to secretion of placental hormones in pregnancy, antagonizes insulin. Blood glucose level enhances in the patient suffering from GD, which also associated with type 2 diabetes, fetal abnormalities, and high blood pressure [2]. Therapeutic interventions available for the management of GD majorly focus on the regulation of blood glucose, which alone incompatible to effective treatment and thus need to develop alternative therapy for the management of it. Recent report evident that dysregulation of insulin signalling in GD occurs due to chronic inflammation, contribute in development of insulin resistance [3]. Phosphorylation of insulin receptor substrate-1 activated by TNF-α, which alter the insulin signalling [4]. Moreover, parameters of inflammatory pathway such as NF-κB and TLR4 also contribute in the development of GD [5]. Thus, targeting inflammatory pathway could be used for the management of GD.

Supportive and alternative therapy gain the importance for the effective management of several diseases including diabetes and GD. Bergaptol is abundantly present in citrus plant also known as furocoumarin [6]. Reported investigation shows anti-inflammatory and antitumor activity of bergaptol [7,8], it reported to show protective effect against neuroinflammation and neuronal injury by regulating JAK2/STAT3/p65 pathway [9]. Moreover, bergaptol has shown antioxidant, anti-osteoporosis and antihyperlipidemic property on the basis of reduction of oxidative stress and inflammatory mediators [10–12]. Plants such as *Ficus religiosa, Ducrosia anethifolia Boiss, lemon peel and Exocarpium citri Grandis* contain bergaptol has shown promising effect against hyperlipidaemia and diabetes, promotes lipolysis [13–17]. These evidences support for the assessment of bergaptol effect on GD.

## Material and Methods

### Gestational diabetes induction

Animal protocol was approved by institutional animal ethical committee of Chengdu University of Traditional Chinese Medicine, China (650/05/C/CPCSEA/09). Healthy female SD rats (180–210 g) were housed under controlled condition as per guideline. Animals were feed with high fat diet for the duration of eight-week [18], Female rats were mated with male rats by keeping them in cage at 2:1 ratio and presence of sperm in vagina confirm by observing it in microscope, the day is counted as day 0 of protocol. Later STZ (25 mg/kg) was injected i.p. after 12 h of fasting [19], blood glucose was estimated for 72 h. Rats with 200 mg/dl of blood glucose considered as GD.

Pregnant female rats with GD were separated in to five different groups (n=6/group) such as control group, GD group, bergaptol 20 and 40 mg/kg group receives bergaptol 20 and 40 mg/kg [9] for the duration of 18 days. Standard (STD) group receives metformin 100 mg/kg, p.o. for the duration of 18 days. Blood was withdrawn from retro orbital pluxes for the estimation of biochemical parameters at the end of protocol.

### Insulin tolerance and glucose level test

Insulin tolerance and glucose level test were performed on GD rats. In OGTT, glucose was administered i.p. after 6 h of fasting at a dose of 2 g/kg and blood was withdrawn from the retroorbital sinus at 30, 60, 90 and 120 min after the administration of glucose, blood glucose level was estimated by autoanalyzer. In ITT, insulin (0.75 U/kg) was injected i.p. in rat by estimating the level of blood glucose at 30, 60, 90 and 120 min after insulin administration.

### Estimation of lipid profile, insulin and C-reactive protein level

Lipid profile level was estimated in the serum with autoanalyzer. Level of insulin in blood was estimated using ELISA kit as per the direction given in the kit. C-reactive protein level was also estimated in the blood using ELISA kit.

### Assessment of inflammatory cytokines and oxidative stress parameters

Inflammatory cytokines such as IL-1β, IL-6, and TNF-α were estimated in the serum of GD rats with ELISA reader (Thermo Fisher Scientific Inc, USA) as per the direction given in its kit (ARG83004; arigoPLEX®, Taiwan).

Parameters of oxidative stress such as MDA, SOD and GSH were estimated in the pancreatic tissue of GD rats. Okhawa *et al*., method was used to estimate the MDA level, biological samples were reacted with thiobarbituric acid and absorbance was estimated at a wavelength of 532 nm [20]. Misra and Fridovich reported method was used to estimate activity of SOD, biological samples were treated with epinephrine and absorbance was estimated at 480 nm to observe the alteration in activity of SOD [21]. Glutathione (GSH) level was estimated by treating 0.2 ml of tissue homogenate with DTNB (0.1 ml) and phosphate buffer (0.8 ml), absorbance was determined at 412 nm [22].

### qRT-PCR analysis

Expression of INSR, NF-κB, Akt and GSK-3β were assessed in the tissue homogenate of GD rats by Quantitative real-time PCR analysis. RNA was extracted out from the tissue homogenate with TRIzol reagent. Bio-red iScript cDNA synthesis kit was used to synthesize the cDNA as per the direction given manufacturer of kit. qPCR study was performed to estimate the expression, as SYBR Green Select PCR Master Mix kit (9L), in each combination with cDNA (2L) and each primer (1L). List of primers used in the study shown in Table 1. Expression were performed in conditions like 95 °C, 95 °C, and 50 °C temperature, the first hold for pre-saturation, denaturation, and annealing holds was noted at 15 min, 40 cycles in 10 s and 15 s, respectively. Thereafter reaction was hold finally at 72 °C for 60 s. Negative control was considered as separate response which do not includes cDNA in reaction. ΔΔCT values was analysed the folds of increase in gene expression by comparing the value of ΔCT between experimental and control samples.

#### In silico

##### Molecular targets of bergaptol against Gestational Diabetes/Diabetes: Network pharmacology

Structural information of bergaptol was taken from the PubChem and protein targets of it were searched from the SwissTarget database (http://swisstargetprediction.ch). Gene contributes in the development of Gestational Diabetes, which we took type 2 diabetes from DisGeNET database (https://disgenet.com).

Protein targets overlap between type 2 diabetes and bergaptol targets with Veeny 2.1. Common targets were applied to the multiple protein section of STRING database (https://string-db.org) to assess the PPI network, confidence score ≥0.7 was used to create the network. Moreover, Gene Ontology (GO) and KEGG enrichment analysis was performed to assess the pathway type 2 diabetes by SR plot (https://www.bioinformatics.com.cn).

##### Assessment of bergaptol interaction with INSR, NF-κB, Akt and GSK-3β proteins: Molecular docking study

PubChem compound database (https://pubchem.ncbi.nlm.nih.gov/) was used to retrieve the Metergoline structure. Selected protein i.e. INSR (PDB: 5h8h), NF-κB, Akt and GSK-3β was retrieved from the Protein Data Bank (PDB) (http://www.rcsb.org/pdb). Docking study was performed with Autodock Vina software, structure of 5h8h was prepared in Discovery Studio Visualizer v20.1.0.19295 software, Dassault Systèmes: San Diego, CA, USA, 2020 by removing Hetatms from the pdb. Polar-H atoms was added to the structure in autodock Vina software, grid for the binding of ligand was selected with Autogrid4. Binding energy and H-bond formation with amino acid of protein was assessed with Autodock Vina software.

##### Statistical analysis

Data are represented as mean ± SEM (n=6). Analysis of variance (ANOVA) was used for the comparison between different groups followed by *post hoc* Bonferroni test with GraphPad Prism, version 9.2 (GraphPad Software Inc., Boston, MA, USA). p<0.05 was considered statistically significant.

## Results

### Bergaptol ameliorate body weight

**Fig. 1:**
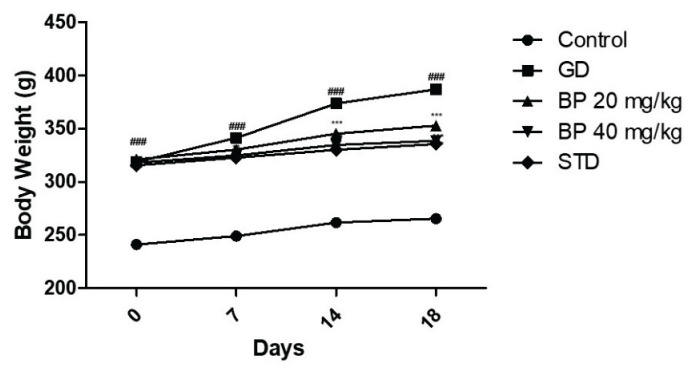
Bergaptol attenuates body weight of GD rats on 0^th^, 7^th^, 14^th^ and 18^th^ day of protocol. Mean ± SEM (n=6); ^###^ p<0.001 than control group; *** p<0.001 than GD group.

Body weight was observed in bergaptol treated GD rats on 0^th^, 7^th^, 14^th^ and 18^th^ day of protocol as shown in Figure 1. There was improvement in the body weight of all the group than control group of rats on the 0^th^ day of protocol, this improvement in body weight of all the group rats occur due to feed of high fat diet for the duration of eight weeks excluding control group which receives normal diet. Body weight was increased significantly (p<0.01) in GD group than control group of rats on 7^th^, 14^th^ and 18^th^ day of STZ administration. Treatment with bergaptol and STD drug attenuates increased body weight of GD rats on 7^th^, 14^th^ and 18^th^ day of protocol.

### Bergaptol ameliorates insulin intolerance and glucose level

**Fig. 2:**
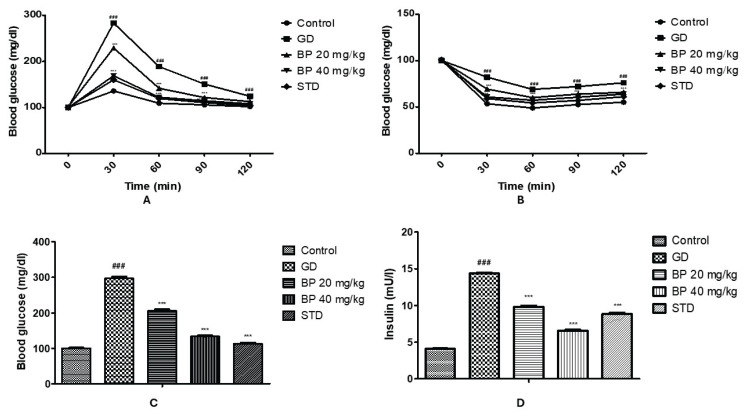
Bergaptol ameliorates insulin and blood glucose level in GD rats. (**A**) Glucose tolerance test; (**B**) Insulin intolerance test; (**C**) Assessment of blood glucose level; (**D**) Assessment of insulin level. Mean ± SEM (n=6); ^###^ p<0.001 than control group; *** p<0.001 than GD group.

Level of blood glucose and insulin was estimated in bergaptol treated GD rats as shown in Figure 2A–D. Glucose tolerance was performed by estimating blood glucose level at 0, 30, 60, 90 and 120 min after administration of glucose. Level of glucose was significantly enhances in GD group than control group rats, which was reversed in bergaptol treated group (Fig. 2A). Insulin intolerance test also shows less reduction in level of blood glucose at 30, 60, 90 and 120 min after the administration of insulin in GD group than control group, bergaptol treated group shows significant reduction in glucose level than GD group of rats (Fig. 2B). Moreover, level of blood glucose and insulin was estimated at the end of protocol, Treatment with bergaptol ameliorates altered level of blood glucose and insulin in GD group of rats (Fig. 2C–D).

### Bergaptol ameliorates altered level of total cholesterol, triglyceride and HDL-cholesterol

**Fig. 3:**
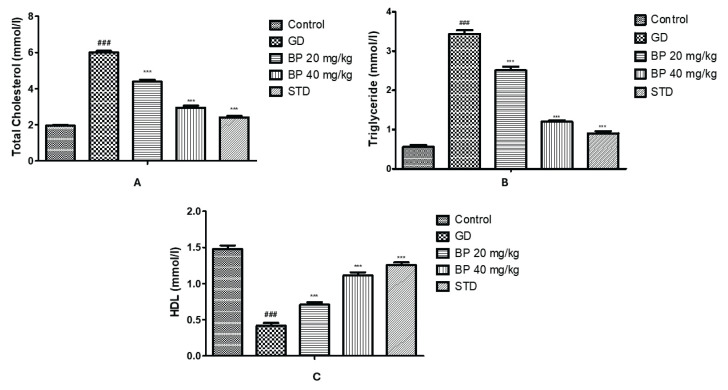
Bergaptol ameliorates the altered level lipid profile in the serum of GD rats. (**A**) Total cholesterol; (**B**) Triglyceride; (**C**) High density lipoprotein. Mean ± SEM (n=6); ^###^ p<0.001 than control group; *** p<0.001 than GD group.

Total cholesterol and triglyceride level were observed to be enhanced to 5.99±0.10 and 3.43±0.09 mmol/l in the serum of GD group than control group (TC: 1.95±0.05 mmol/l and TG: 0.55±0.05 mmol/l) respectively. Treatment with bergaptol significantly reduces the level of TC (2.94±0.11 mmol/l) and TG (1.19±0.03 mmol/l) than GD group of rats. Moreover, HDL-cholesterol level reduces up to 0.41±0.03 mmol/l in GD group than control group (1.48±0.05 mmol/l) of rats, which was enhanced up to 1.11±0.04 mmol/l in bergaptol treated GD rats (Fig. 3A–C).

### Bergaptol ameliorates altered level of inflammatory mediators

**Fig. 4:**
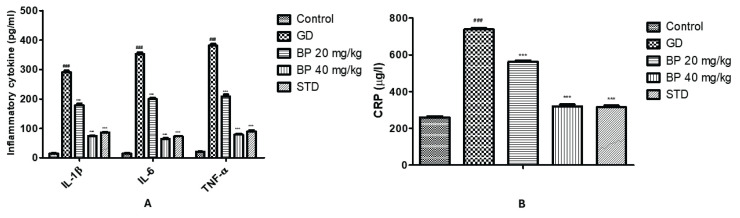
Bergaptol ameliorates the altered level of inflammatory mediators in the serum of GD rats. (**A**) Inflammatory cytokines level in the serum of GD rats; (**B**) C-reactive protein level in the serum of GD rats. Mean ± SEM (n=6); ^###^ p<0.001 than control group; *** p<0.001 than GD group.

Factors involved in the inflammation were estimated in the serum of bergaptol treated GD rats shown in Figure 4A, B. Cytokines like IL-1β, IL-6 and TNF-α were enhanced up to 291.36±4.68, 352.5±5.53 and 380.78±6.67 pg/ml respectively in the serum of GD group than control group (IL-1β: 14.19±1.55 pg/ml; IL-6: 15.03±1.54 pg/ml and TNF-α: 20.86±0.94 pg/ml). Treatment with bergaptol significantly reduces the level of IL-1β (74.15±2.39 pg/ml), IL-6 (64.6±2.64 pg/ml) and TNF-α (78.91±3.69 pg/ml) in GD rats (Fig. 4A). CRP was enhanced significantly (p<0.001) up to 738.39±6.47 μg/l compared to control group (260.34±6.7 μg/l), which was reduced in bergaptol treated GD rats up to 320.29±10.36 μg/l as shown in Figure 4B.

### Bergaptol ameliorates altered level of oxidative stress parameters

**Fig. 5:**
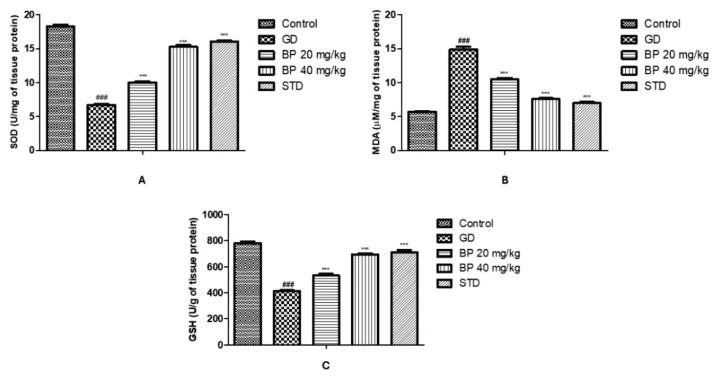
Bergaptol ameliorates the altered level of oxidative stress parameters in the tissue homogenate of GD rats. (**A**) SOD; (**B**) MDA; (**C**) GSH. Mean ± SEM (n=6); ^###^ p<0.001 than control group; *** p<0.001 than GD group.

Parameters of oxidative stress were estimated in the pancreatic tissue homogenate of bergaptol treated GD rats. Activity of SOD and GSH was reduced up to 6.73±0.12 U/mg and 411.85±8.23 U/g of tissue protein respectively in GD group than control group rats, which was improved up to SOD: 15.27±0.26 U/mg and GSH: 695.33±13.29 U/g of tissue protein in bergaptol treated GD rats. However, level of MDA (14.89±0.39 μM/mg) was enhanced in GD group than control group (5.72±0.08 μM/mg). Treatment with bergaptol significantly reduces MDA level up to 7.58±0.15 μM/mg in the pancreatic tissue of GD rats (Fig. 5).

### Bergaptol ameliorates altered expression of INSR/NF-κB/Akt/GSK3β

**Fig. 6:**
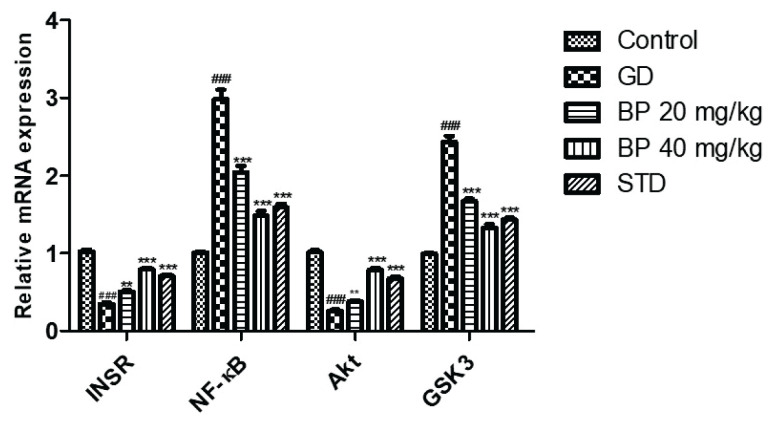
Bergaptol ameliorates the altered mRNA expression of INSR, NF-κB, Akt and GSK-3β in the tissue homogenate of GD rats. Mean ± SEM (n=6); ^###^ p<0.001 than control group; *** p<0.001 than GD group.

Assessment of bergaptol effect was observed on the mRNA expression of INSR, NF-κB, Akt and GSK-3β in the tissue homogenate of GD rats as shown in Figure 6. mRNA expression of INSR and Akt were observed to be reduced and mRNA expression of NF-κB and GSK-3β were significantly enhanced in the tissue homogenate of GD group than control group of rats. Treatment with bergaptol ameliorates the altered mRNA expression of INSR, NF-κB, Akt and GSK-3β in the tissue homogenate of GD rats.

### Insilico assessment of potential targets and pathways of bergaptol against type 2 diabetes: Network Pharmacology

Potential targets of bergaptol was assessed from the database for therapeutic potential against insulin resistance i.e. type 2 diabetes, which clinically matches to GD. Bergaptol (PubChem CID: 5280371; Molecular weight: 202.16 g/mol) was assessed from PubChem NCBI site to identify simple (C1=CC(=O)OC2=CC3=C(C=CO3) C(=C21)O) and CAS number is 486-60-2. Prediction of molecular targets of bergaptol was assessed from SwissTarget database and 100 protein targets were identified, with ≥0.1 probability for the binding of bergaptol, which is given below in Table 2.

**Fig. 7:**
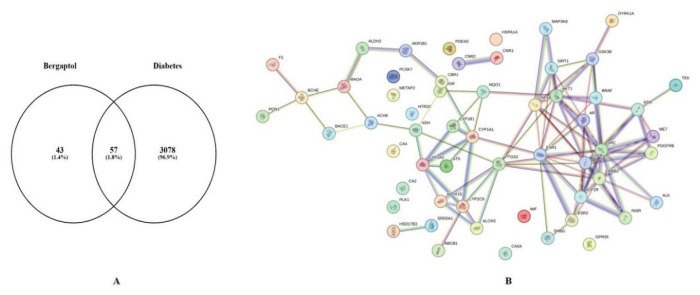
Construction of common targets of interaction of bergaptol and type 2 diabetes along with assessment of network of these targets PPI. (**A**) Identification of intersection targets between bergaptol and type 2 diabetes. (**B**) 3D form of visualization of protein-protein interaction (PPI) network of the 57 targeted genes type 2 diabetes. STRING Web server was used to construct the PPI network with threshold of 0.7.

Gene involved in the development of type 2 diabetes was assessed from DisGeNET database and it was observed that 3078 number of gene relate to it. Mapping of molecular targets of bergaptol with identified gene was performed with Veeny 2.1. and 57 common targets were identified, which shows overlapping of molecular targets of bergaptol and gene involved in the development of type 2 diabetes (Fig. 7A). These mapped genes were used to construct the PPI network with the confidence of 0.7 using STRING database online server. There are 57 nodes, 106 edges of average nodes degree 3.72 and avg. local clustering coefficient: 0.467 as shown in Figure 7B.

**Fig. 8:**
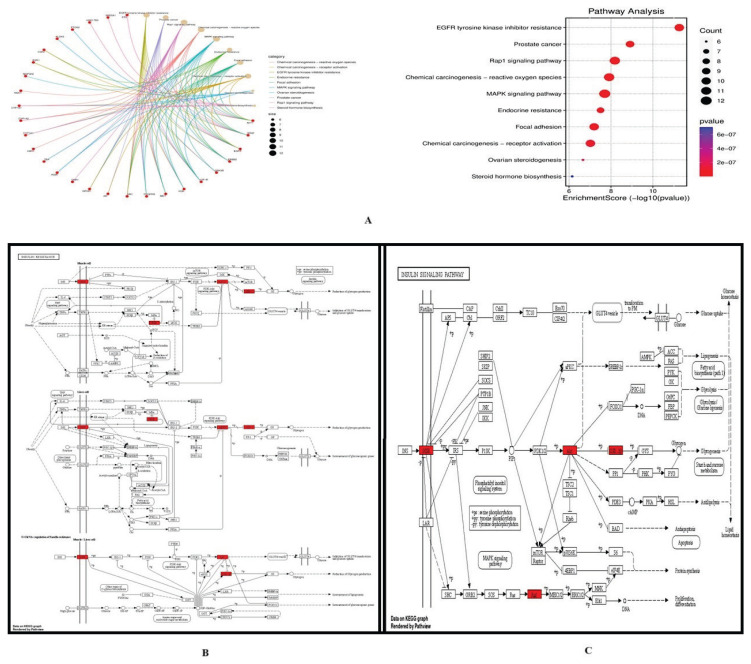
Assessment of fundamental mechanism of bergaptol for the management of type 2 diabetes through network pharmacology. (**A**) GO analysis of the target proteins of bergaptol on type 2 diabetes; (**B**) Kegg Pathway for insulin resistance formation of bergaptol against type 2 diabetes; (**C**) Kegg Pathway for insulin signalling formation of bergaptol against type 2 diabetes.

GO analysis and Kegg pathway was performed on potential targets of bergaptol for the management of type 2 diabetes. Specifically, 47 targets were observed in GO analysis, observation shows INSR, NF-κB, Akt, GSK-3 and Raf, which regulates insulin signalling and insulin resistance pathway, these pathways contribute in the development of specifically type 2 diabetes (Fig. 8A–C). Identified targets such as INSR, NF-κB, Akt, GSK-3 and Raf from GO analysis were further assessed for binding with bergaptol by molecular docking study.

### Assessment of bergaptol interaction with INSR, NF-κB, Akt and GSK-3β protein: Molecular docking study

**Fig. 9:**
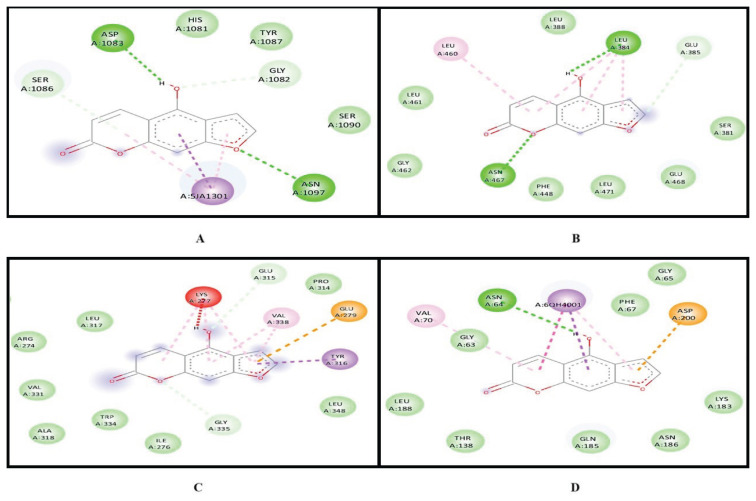
Assessment of bergaptol interaction with INSR, NF-κB, Akt and GSK-3β protein by molecular docking study. (**A**) Interaction of bergaptol with INSR protein; (**B**) Interaction of bergaptol with NF-κB protein; (**C**) Interaction of bergaptol with Akt protein; (**D**) Interaction of bergaptol with GSK-3β protein.

Hydrogen bond formation with protein residue and binding energy was assessed in protein-ligand complex as shown in Table 3. We observed that Bergaptol binds with INSR, NF-κB, Akt and GSK-3β proteins with maximum energy at binding energy of −6.1, −6.6, −8.2 and −6.9 kcal/mol respectively and interacted with four protein residue INSR (ASP-A:10833, ASN-A: 1097), NF-κB (LEU-A:384, ASN-A:467), Akt (GLU-A:315, GLY-A:335) and GSK-3β (ASN-A:64) proteins. Moreover, 2D molecular structure of ligand-protein complex is represented in Figure 9A–D).

## Discussion

Gestational diabetes resembles the clinical pathophysiology to type 2 diabetes, as insulin resistance and glucose tolerance involved in the pathogenesis of it. Prevalence rate is 14 %, which predicted to be gradually enhancing in developing countries [23]. Pregnancy is a specific situation to use of selected drugs including in the regulation of blood glucose too. Thus, there is need of development of alternative medicine for the management of GD and given report efforts the same by evaluating beneficial effect of bergaptol against GD.

Weight gain is specifically characterized for the development of GD, as it is also one of the major causes of several disorder including diabetes specifically GD and type 2 diabetes [24]. Oral hypoglycaemic drugs i.e. metformin reduces the body weight while managing blood glucose in diabetic patients [25]. It is also evident that reduction of obesity reduces the quantity of white adipose tissue, which reduces body weight that also involved in the improvement of sensitivity of insulin receptor (IR) too [26]. Insulin resistance occurs due to loss of sensitivity of its receptor, which causes reduction in the formation of GLUT, reduces utilization of glucose, which promotes the level of blood insulin in GD and type 2 diabetes [27]. Drugs used for the management of GD promotes the sensitivity of IR, which enhances the utilization of glucose and reduces the blood glucose level [28]. There are several derivatives of furanocoumarins has several potential therapeutic potentials on the regulation of blood glucose and obesity management [29], however bergaptol has not investigated for it yet. Bergaptol has shown anti-inflammatory, antioxidant and antihyperlipidemic properties [7,9,10], which involve in the weight gain and regulation of insulin sensitivity. Given investigation depict that body weight of bergaptol treated rats were significantly reduced than GD group rats. There was significant reduction in the level of blood glucose (GTT and at the end of protocol) and insulin level in bergaptol treated GD rats. In insulin intolerance test, level of blood glucose significantly improved in bergaptol treated group than GD group of rats. Metformin reduces blood glucose, body weight and improves insulin sensitivity in diabetic patients and data of study also suggest the same. Bergaptol regulates these parameters similar to metformin in diabetic rats.

Dyslipidaemia is one of the major clinical features associated with metabolic disorders including diabetes, which involve in the development of diabetic complications. Glucose utilisation reduces in GD which alters the lipid level due to dysregulation of metabolic pathway [30]. Total cholesterol and triglyceride level enhances in the blood in the patients suffering from GD, moreover HDL reduces in patients suffering from GD [31]. Bergaptol reported to be bergaptol is present in essential oils of citrus peel call as furanocoumarins [6], which shows antihyperlipidemic effect by promoting lipolysis and reducing the cellular uptake of lipid [32]. These effect of furanocoumarins i.e. bergaptol contribute in the regulation of lipid level and our report also support that treatment with bergaptol attenuates altered lipid level in GD rats.

Metabolism alteration enhances free radical level by promoting the production of cellular level of superoxide anions, which increases oxidative stress. MDA is a metabolic product of lipid by the process of lipid peroxidation, injury to the cell membrane, level of MDA enhances in GD [33]. SOD and GSH activity involved in the reduction of superoxide anions in the cells, which also altered in GD. These parameters of oxidative stress contribute in the dysregulation of cellular function, enhances inflammation process as NF-κB is reported to be activated by cytokine and oxidative stress which activate autoimmunity [34]. Further activation of NF-κB reduces the expression of Akt that enhances the expression of GSK-3β. This altered expression of gene reduces the production GLUT, which reduces the utilization of glucose lead to increase in the level of glucose [35]. Moreover, autoimmunity altered the sensitivity of IR, reduced insulin sensitivity decreases the expression of GLUT.

Drugs used for the management of GD effect-tively reduces oxidative stress and inflammatory cytokines, which also reported to be regulate the inflammatory pathway and thereby enhances the utilization of glucose [36]. Data of our report reveals that treatment with bergaptol ameliorates the altered level of inflammatory mediators and oxidative stress parameters in GD rats. *In vivo* study also suggest expression of INSR, NF-κB, Akt and GSK-3β regulated in the bergaptol treated GD rats. Moreover, network pharmacology study involve common targets of bergaptol and type 2 diabetes, which was further applied for gene ontology suggested INSR, NF-κB, Akt and GSK-3β targets for the insulin signalling and resistance both. These targets interacted with bergaptol ligand for the management of GD, as it resembles in pathogenic aspects of type 2 diabetes. Moreover, docking study also confirm the effective interaction of bergaptol with INSR, NF-κB, Akt and GSK-3β targets.

## Conclusions

In conclusion, data of investigation suggest that bergaptol reduces blood glucose level in GD, as it reduces parameters of oxidative stress and inflammatory mediators by regulating INSR/NF-κB/Akt/GSK-3β pathway. Moreover, data of *in silico* also supports that bergaptol interact with these proteins and thereby regulates the insulin sensitivity and insulin signalling pathway. These evidences support that bergaptol could be used for the management of GD clinically. However, molecular mechanism along with chronic toxicity study and teratogenicity study of bergaptol needed to assess for the better healthcare management.

## Figures and Tables

**Table 1:** Primer details.

Sr. No.	Primer	Forward	Reverse
1	INSR	AGATGAGAGGTGCAGTGTGGCT	GGTTCCTTTGGCTCTTGCCACA
2	NF-κB	GCTGCCAAAGAAGGACACGACA	GGCAGGCTATTGCTCATCACAG
3	Akt	GAGATGGATGCGTCTACAACCC	TCCACTTGCCTTCTCTCGAACC
4	GSK-3β	GAGCCACTGATTACACGTCCAG	CCAACTGATCCACACCACTGTC

**Table 2:** Protein targets of bergaptol screen from SwissTarget database.

XDH	CA5B	PTK2	NQO1	GPR35	ALDH2	CA5A	ALOX15
ESR1	HSD17B3	KDR	ALK	ERBB2	CDK5R1 CDK5	BACE1	AR
ESR2	GSR	PLK1	AXL	AKR1B1	STS	MAP3K8	MIF
CA12	CYP1A2	CA6	DYRK1A	CCND1 CDK4	MAOB	BRAF	PDE4D
CA9	MAOA	CA14	HTR2C	PDGFRB	APEX1	EPHB4	ABCB1
CA7	CA2	CSNK2A1	ADORA2A	FLT4	CYP1B1	HSPA1A	ERN1
CA13	CA1	MET	METAP2	IGF1R	PIM1	NUAK1	PTGS2
ACHE	TNNC1 TNNT2 TNNI3	CA4	FYN	INSR	ERCC5	SQLE	SLC16A3
NFKB1	SRD5A1	PLK4	LCK	CDK2 CCNA1 CCNA2	FEN1	FGR	OPRK1
ALOX5	CA3	TEK	F2	AURKB	CNR1	LYN	SIRT1
CBR1	AKR1C3	AKT1	GRK6	GSK3B	CNR2	KCNA5	PRKDC
EGFR	AKR1C1	AURKA	BCHE	SRC	PON1	KCNA3	CYP1A1
PCSK7	CYP2C9	DAO	SHBG				

**Table 3:** Ligand-protein interaction assessed for the estimation of H-bond formation with protein residue and binding energy.

Sr. No.	Ligand	Protein	Binding Energy (kcal/mol)	Amino Acid
1	Bergaptol	INSR	−6.1	ASP-A:10833, ASN-A: 1097
2		NF-κB	−6.6	LEU-A:384, ASN-A:467
3		Akt	−8.2	GLU-A:315, GLY-A:335
4		GSK-3β	−6.9	ASN-A:64
